# 

**Published:** 1914-12

**Authors:** 


### The Library of the Bristol Medico-Chirurgical Society.

### NINETY-FOURTH LIST OF BOOKS.

*The following books have been received since the publication of the List in September.*

*November 30th,* 1914.

| | | |
|---|---|---|
| **Bainbridge and J. A. Menzies, F. A.** | *Essentials of Physiology* .. .. | 1914 |
| **Calot, F.** .. .. | *Indispensable Orthopædics* (Translated from 6th French Edition by A. H. Robinson and L. Nicole) .. .. .. .. .. .. .. .. | 1914 |

**Cunningham [D. J.]** *Manual of Practical Anatomy* (Revised and edited by A. Robinson) Vol. II. 6th Ed. 1914
**Despard, L. L.** .. *Text-book of Massage* .. .. .. 2nd Ed. 1914
**Elmslie, R. C.** .. *Remedial Exercises in Schools and Clinics* .. 1914
**Fitzwilliams, D. C. L.** *A Nursing Manual* .. .. .. .. .. .. .. 1914
**Friel, A. R.** .. .. *Obiter Scripta Throat, Nose and Ear* .. .. 1914
**Gould, Sir A. P.** .. *Surgical Diagnosis* .. .. .. .. 4th Ed. 1914
**Goulston, A.** .. .. *Cane Sugar and Heart Disease* .. .. .. 1914
**Hayes, R.** .. .. *Aachen Treatment of Syphilis* .. .. .. .. .. 1914
**Lejars, F.** .. .. *Urgent Surgery* (Translated from 7th French Edition by W. S. Dickie) Vol. I. 3rd English Ed. .. .. .. .. .. .. .. 1914
**Menzies, F. A. Bainbridge and J. A.** *Essentials of Physiology* .. 1914
**Oliver, Sir T.** .. *Lead Poisoning* .. .. .. .. .. .. .. .. 1914
**Paton, D. N.** .. .. *Essentials of Human Physiology* .. 4th Ed. 1914
*Pharmacopœia, The British* .. .. .. .. .. .. .. .. .. .. 1914
**Quain, [J.]** .. .. *Elements of Anatomy* .. Vol. II. Part II. 11th Ed. 1914
**Sutherland, G. A.** .. *The Heart in Early Life* .. .. .. .. .. .. 1914

TRANSACTIONS, REPORTS, JOURNALS, ETC.

American Ophthalmological Society, Transactions of the Vol. XIII. Part III. 1914
Archives of the Röntgen Ray .. .. .. .. Vol. XVIII. 1913–14
Collected Studies from the Laboratories of Health, New York Vol. VII. 1912–14
Hospital Reports, Episcopal .. .. .. .. .. .. .. .. Vol. II. 1914
Medical Officer, The .. .. .. .. .. .. .. .. .. Vol. XI. 1914
Ophthalmological Transactions .. .. .. .. .. Vol. XXXIV. 1914
Progressive Medicine .. .. .. .. .. .. .. .. .. Vol. III. 1914
Revue Hebdomadaire de Laryngologie .. Tom. XXXV., Vol. I. 1914
Royal Academy of Medicine in Ireland, Transactions of the Vol. XXXII. 1914
Zentralblatt für Chirurgie .. .. .. .. .. .. .. .. Bd. I. for 1914
Zentralblatt für Gynäkologie .. .. .. .. .. .. Bd. I. for 1914
Zentralblatt für innere Medizin .. .. .. .. .. .. Bd. I. for 1914

### MEETINGS OF SOCIETIES.

### Bristol Medico-Chirurgical Society.

*Annual Meeting, October 14th, 1914.*

After a vote of thanks to the retiring President, Dr. P. Watson-Williams, had been passed, Dr W. H. C. Newnham took the chair, and gave an introductory address (*see* p. 257). Later a cordial vote of thanks was given to Dr. Newnham for his able and interesting address.

### Local Medical Notes.

**University of Bristol.**—Students of the University have recently passed the following examinations :—

Conjoint Examining Board of England.—*Medicine :* J. W. Gilbert, G. S. Terry, B. G. Derry. *Surgery :* Muir-Foster. *Midwifery :* W. K. A. Richards, C. N. Vaisey, H. R. W. Husbands. *Practical Pharmacy :* A. G. Morris.

Licence in Dental Surgery, R.C.S. England.—*Chemistry:* B. Leathlean. *Physics :* I. Jacobs. *Mechanical Dentistry :* E. F. Vowles, S. W. A. Davis, F. L. Fox. *Dental Metallurgy :* E. F. Vowles, W. G. Milton. *Final Examination, Part I :* W. E. Drummond ; *Part II :* S. V. Shrimpton.

Royal Navy.—At the recent examination of candidates for entry as Surgeons, Mr. H. B. Parker, M.B., a former Bristol student, secured first place with 2,488 marks, and was awarded the gold medal.

R.A.M.C.—B. J. L. Fayle, R. B. Johnson, R. F. Bolt, G. M. Campbell, H. J. Orr-Ewing, H. W. Goodden, R. S. Statham, C. J. D. May, C. C. Harrison, A. G. Bodman, C. Kingston.

R.A.M.C. (T.).—F. T. Boucher, H. J. D. Smythe, R. I. Dacre.

### APPOINTMENTS.

Professor James Swain, M.S., M.D., has been appointed Consulting Surgeon to the Forces serving in the Southern Command, with the rank of Lieut.-Colonel, R.A.M.C.

W. A. Reynolds, M.B., Ch.B., has been appointed House Surgeon at Sheffield Royal Infirmary.

G. H. Griffiths, M.R.C.S., has been appointed House Surgeon at Cheltenham General Hospital.

J. Cecil Clayton, M.R.C.S., L.R.C.P., has been appointed Anæsthetist to the All Saints' Hospital for Genito-Urinary Diseases, Victoria ; to the Royal Ear Hospital, London ; and to Hendon Cottage Hospital.

### ACKNOWLEDGMENT.

Lieut.-Col. A. W. Prichard desires to take advantage of the *Bristol Medico-Chirurgical Journal* to cordially thank those members of the profession who were asked by Dr. Devis and himself to subscribe to a present for the 3rd South Midland Field Ambulance, now mobilised for active service. Thirty shirts and a hundred and eighty long and thick mufflers were sent on November 12th, and the O.C., Lieut.-Col. Young, desired that his appreciation of the gift should be communicated to those who subscribed.

### INDEX.

Analgesia, Local—A. L. Flemming, 193.
Anaphylaxis, 154.
Aneurysm, The simulation of aortic, by some other diseases—Dr. C. Coombs, 26.
Animal extracts in therapeutics, 249.
Arterio-sclerosis, Diet in, 218.
Arterio-sclerosis, Hyperpiesis and—Dr. C. W. J. Brasher, 41.

Bardswell, Dr. N.—Mixed infections in pulmonary tuberculosis, 125.
Bone pegging, Auto-plastic intra-medullary, in fractures—C. F. Walters, 139.
**Books, Reviews of**—
Achondroplasia—Dr. M. Jansen, 234.
Adams, Dr. P. H.—Pathology of the eye, 84.
Adamson, Dr. H. G.—Modern views upon skin eruptions, 86.
Allen, Dr. R. W.—Vaccine therapy, 87.
Anæsthetics—Dr. D. W. Buxton, 237; Sir F. W. Hewitt, 79; Dr. J. F. W. Silk, 237.
Anatomy, Companion to manuals of practical—E. B. Jamieson, 173.
Anatomy, Cunningham's practical, 173.
Appendicitis—E. Owen, 230.
Arteriosclerosis—Dr. L. F. Bishop, 307.
Bandaging, Elements of—Dr. W. Rankin, 73.
Bandaging, Pye's elementary, 83.
Barnetuberkulose, Bakteriologische studier over—A. de Besche, 84.
Barnetuberkulosen's klinische initialformer, Diagnosen av—H. G. Gade, 235.
Bayly, H. W.—Clinical pathology of syphilis, 311.
Bennett, Dr. R. A.—Plain rules for the use of tuberculin, 174.
Bennett, R. R.—Materia medica and pharmacy, 83.
Bernstein, Dr. J. M.—Applied pathology, 164.
Besche, A. de—Bakteriologische studier over barnetuberkulose, 84.
Bidwell, L. A.—Minor surgery, 82.
Bishop, Dr. L. F.—Arteriosclerosis, 307.
Blum, Dr. V.—Renal diagnosis, 166.
Bourne, Dr. A. W.—Synopsis of midwifery, 76.
British journal of surgery, The, 235.
Bruce, O.—Lectures on tuberculosis for nurses, 82.
Bruce, Dr. W.—Sciatica, 82.
Burghard, Sir W. W. Cheyne and F. F.—Surgical treatment, 166.

**Books, Reviews of** (*continued*)—
Buxton, Dr. D. W.—Anæsthetics, 237.
Cammidge, Dr. P. J.—The fæces of children and adults, 313.
Catalogue (Index-) of the library of the Surgeon-General's office, United States army, 81, 175.
Cheyne and F. F. Burghard, Sir W. W.—Surgical treatment, 166.
Chaplin, Dr. A.—Thomas Shortt, 314.
Choyce, C. C.—System of surgery, 229.
Chundra, J. L.—Laws of sexual philosophy, 80.
Clinical medicine, Studies in—Dr. C. O. Hawthorne, 85.
Cole, Dr. R. H.—Mental diseases, 168.
Colitis, Chronic—Drs. G. Herschell and A. Abrahams, 233.
Cotar, Dr. C.—The mineral waters of Vichy, 80.
Coxa vara—R. C. Elmslie, 86.
Cuff, I. Stewart and Dr. H. E.—Practical nursing, 238.
Cunningham's manual of practical anatomy, 173.
Darier, Dr. A.—Ophthalmic therapeutics, 77.
Dental diseases in relation to public health—J. S. Wallace, 176.
Diagnosis, Tabular—Dr. R. W. Leftwich, 311.
Dreams, The interpretation of—Dr. S. Freud, 72.
Dührssen, Dr. A.—Geburtshilfliches vademekum, 172.
Electricity, Medical—Dr. J. D. Harris, 312 Dr. H. L. Jones, 311.
Elliot, Dr. R. H.—Sclero-corneal trephining in glaucoma, 84.
Elmslie, R. C.—Coxa vara, 86.
Eye, Diseases of the—M. S. Mayou, 78; Sir H. R. Swanzy, 78.
Eye, Microscopic examination of the—R. Greeff, 171.
Eye, Pathology of the—Dr. P. H. Adams, 84.
Fæces of children and adults—Dr. P. J. Cammidge, 313.
Fernie, Dr. W. T.—Our outsiders, 312.
Finzi, Dr. N. S.—Radium therapeutics, 76.
Fischer, M. H.—Zur behandlung der nephritis, 170.
Fraser, Dr. E. T.—Manual of immunity, 74.
French, Dr. H.—Medical laboratory methods and tests, 82.
Freud, Dr. S.—The interpretation of dreams, 72.
Gade, H. G.—Diagnosen av barnetuberkulosen's klinische initialformer, 235.
Gas poisoning—Drs. J. Glaister and D. D. Logan, 308.
Geburtshilfliches vademekum—Dr. A. Dührssen, 172.

**Books, Reviews of** (*continued*)—

Glaister and D. D. Logan, Drs. J.—Gas poisoning, 308.
Glaucoma, Sclero-corneal trephining in—Dr. R. H. Elliot, 84.
Gonorrhœa, Text-book on—Dr. G. Luys, 73.
Greeff, R.—Guide to the microscopic examination of the eye, 171.
Groves, E. W. H.—Synopsis of surgery, 175.
Hæmorrhoids, Treatment of—D. D. Wright, 83.
Harris, Dr. J. D.—Lectures on medical electricity, 312.
Harvard medical school, department of neurology, Reports, 171.
Hawkins-Dempster, H.—Explanatory lectures for nurses, 173.
Hawthorne, Dr. C. O.—Studies in clinical medicine, 85.
Heart, Diseases of the—Dr. J. Mackenzie, 312.
Herman, Dr. G. E.—Diseases of women, 77.
Herschell and A. Abrahams, Drs. G.—Chronic colitis, 233.
Hewitt, Sir F. W.—Anæsthetics, 79.
Howard, R.—The practice of surgery, 230.
Hygiene and public health—Dr. L. C. Parkes, 83.
Insanity, Text-book of—Dr. C. A. Mercier, 309.
Immunity, Manual of—Dr. E. T. Fraser, 74.
Ionic medication—Dr. H. L. Jones, 169.
Jamieson, E. B.—A companion to manuals of practical anatomy, 173.
Jansen, Dr. M.—Achondroplasia, 234.
Jones, Dr. H. L.—Ionic medication, 169; Medical electricity, 311.
Kennedy, J. M.—Nietzsche, 305.
Knott, C. G.—Physics, 170.
Laboratory methods and tests, Medical—Dr. H. French, 82.
Leftwich, Dr. R.W.—Tabular diagnosis, 311.
Logan, Drs. J. Glaister and D. D.—Gas poisoning, 308.
Lugaro, E.—Modern problems in psychiatry, 167.
Luke, Dr. T. D.—Natural therapy, 312.
Luys, Dr. G.—Text-book on gonorrhœa, 73.
McDonagh, J. E. R.—Salvarsan, 77.
Macdonald, Dr. D. M.—The practitioner's practical prescriber, 236.
Mackenzie, Dr. J.—Diseases of the heart, 312.
Materia medica and pharmacy—R. R. Bennett, 83.
Materia medica notes—J. A. Whitla, 236.
Mayou, M. S.—Diseases of the eye, 78.
Medical annual, The, 81, 165.
Mental deficiency—A. F. Tredgold, 235.
Mental deficiency act, Guide to the—J. and S. Wormald, 171.
Mental diseases—Dr. R. H. Cole, 168.
Mercier, Dr. C. A.—Text-book of insanity, 309.
Midwifery, Synopsis of—Dr. A. W. Bourne, 76.
Miles, A. Thomson and A.—Operative surgery, 86.
Mind and its disorders—Dr. W. H. B. Stoddart, 78.
Natural therapy—Dr. T. D. Luke, 312.
"Nauheim" treatment—Dr. L. T. Thorne, 80.
Nephritis, Zur behandlung der—M. H. Fischer, 170.
Nietzsche—J. M. Kennedy, 305.
Nurses, Explanatory lectures for—H. Hawkins-Dempster, 173.

**Books, Reviews of** (*continued*)—

Nursing, Practical—I. Stewart and Dr. H. E. Cuff, 238.
Obstetric aphorisms—Dr. J. G. Swayne, 75.
Old age, The road to a healthy—T. B. Scott, 176.
Ophthalmic therapeutics—Dr. A. Darier, 77.
Outsides, Our—Dr. W. T. Fernie, 312.
Owen, E.—Appendicitis, 230.
Parkes, Dr. L. C.—Hygiene and public health, 83.
Paterson, H. J.—Surgery of the stomach 164.
Pathology, Applied—Dr. J. M. Bernstein, 164.
Philadelphia, Transactions of the College of Physicians of, 81.
Physics—C. G. Knott, 170.
Prescriber, The practitioner's practical—Dr. D. M. McDonald, 236.
Prescribing, Practical—A. H. Prichard, 236.
Prichard, A. H.—Practical prescribing, 236.
Psychiatry, Modern problems in—E. Lugaro, 167.
Pye's elementary bandaging, 83.
Radium therapeutics—Dr. N. S Finzi, 76.
Rankin, Dr. W.—Elements of bandaging, 73.
Renal diagnosis—Dr. V. Blum, 166.
Riviere, Dr. C.—The early diagnosis of tubercle, 173.
Salvarsan—J. E. R. McDonagh, 77.
Sanitary inspector's handbook, The—A. Taylor, 237.
Sciatica—Dr. W. Bruce, 82.
Scott, T. B.—The road to a healthy old age, 176.
Sexual philosophy, Laws of—J. L. Chundra, 80.
Shortt, Thomas—Dr. A. Chaplin, 314.
Silk, Dr. J. F. W.—Modern Anæsthetics, 237.
Skin eruptions, Modern views upon—Dr. H. G. Adamson, 86.
Small-pox, Administrative control of—W. M. Wanklyn, 238.
Smithsonian report, The, 85.
Spiritual healing, 214.
Stewart and Dr. H. E. Cuff, I.—Practical nursing, 238.
Stoddart, Dr. W. H. B.—Mind and its disorders, 78.
Stomach, Surgery of—H. J. Paterson, 164.
Surgery, Minor—L. A. Bidwell, 82.
Surgery, Operative—A. Thomson and A. Miles, 86.
Surgery, Practice of—R. Howard, 230.
Surgery, Synopsis of—E. W. H. Groves, 175.
Surgery, System of—C. C. Choyce, 229.
Surgical treatment—Sir W. W. Cheyne and F. F. Burghard, 166.
Swanzy, Sir H. R.—Diseases of the eye, 78.
Swayne, Dr. J. G.—Obstetric aphorisms, 75.
Syphilis, Clinical pathology of—H. W. Bayly, 311.
Taylor, A.—The sanitary inspector's handbook, 237.
Thomson and A. Miles, A.—Operative surgery, 86.
Thorne, Dr. L. T.—The "Nauheim" treatment, 80.
Treatment, Medical—Dr. I. B. Yeo, 169.
Tredgold, A. F.—Mental deficiency, 235.
Tubercle, The early diagnosis of—Dr. C. Riviere, 173.
Tuberculin, Plain rules for the use of—Dr. R. A. Bennett, 174.
Tuberculosis for nurses, Lectures on—O. Bruce, 82.

**Books, Reviews of** (*continued*)—
Vaccine therapy—Dr. R. W. Allen, 87.
Vichy, Mineral waters of—Dr. C. Cotar, 80.
Wallace, J. S.—Dental diseases in relation to public health, 176.
Wanklyn, W. M.—The administrative control of small-pox, 238.
Whitla, J. A.—Materia medica notes, 236.
Women, Diseases of—Dr. G. E. Herman, 77.
Wormald, J. and S.—Guide to the mental deficiency act, 171.
Wright, D. D.—Treatment of hæmorrhoids, 83.
Yeo, Dr. I. B.—Manual of medical treatment, 169.

Brasher, Dr. C. W. J.—Hyperpiesis and arterio-sclerosis, 41.
Bristol Medico-Chirurgical Society, 93, 187, 317.
Bristol medical societies, Notes on some—Dr. G. M. Smith, 268.
Bristol resurrectionists, Notes on some, 319.
British Medical Association, Aberdeen meeting, 239.
British pharmacopœia, Some impressions of—Dr. O. C. M. Davis, 292.

Carcinoma, Digital percussion and the cardiac sign in—Dr. W. Gordon, 19.
Cardiac sign in carcinoma, Digital percussion and the — Dr. W. Gordon, 19.
Carotid body, Tumours of, 162.
Cataract, Senile, treatment of, 304.
Cell proliferants, 181.
Clarke, Dr. J. M.—Diseases of the lungs associated with the presence of Friedländer's bacillus, 1 ; Report on medicine, 217.
Clinical methods, Demonstrations of, 95.
Coliuria in children, Clinical manifestations and treatment of—Dr. R. G. Gordon, 286.
Colloid metals, 88.
Coombs, Dr. C.—The simulation of aortic aneurysm by some other aortic and cardiac diseases, 26.
Cotton, Dr. W.—The radiographic centroscope and the radiographic episcope, 202.
Cysts in anterior chamber of the eye, 303.

Davis, Dr. O. C. M.—Some impressions of the 1914 B. P., 292.
Diabetes insipidus in a child, 300.
Diabetes mellitus, Blood-sugar in, 297.
Diabetic coma, 299.
Diet in diseases of the heart, vessels and kidneys, 217.

Edgeworth, Dr. F. H.—On the occurrence of tachycardia in association with parenchymatous goitre, 144, 187.
Editorial notes, 177, 239, 315.
Electrical reactions in facial paralysis—Dr. J. P. I. Harty, 55.
Embolism and thrombosis of mesenteric vessels, 68.
Ethyl-hydrocuprein, 303.

Facial paralysis, Electrical reactions in—Dr. J. P. I. Harty, 55.
Femur, Fracture of the neck of the, 300.
Fibroid uterus—Dr. W. H. C. Newnham, 37.
Firth, Dr. J. L.—Reports on surgery, 68, 224.
Flemming, A. L.—Local analgesia in surgical operations, 193.
Fortescue-Brickdale, Dr. J. M.—Therapeutic notes, 88, 181, 249; Report on medicine, 297.
Fracture of the neck of the femur, 300.
Fractures, Autoplastic intra-medullary bone pegging in—C. F. Walters, 139.
Fractures, Exhibition illustrating the operative treatment of, 187.
Fractures, Modern ideas in the treatment of, 187.
Friedländer's bacillus, Diseases of the lungs associated with—Dr. J. M. Clarke, 1.
Frontal sinus operation, intranasal, Two cases of, 95.

Gastric ulcer, Surgical treatment of, 224.
Goitre, The occurrence of tachycardia in association with parenchymatous—Dr. F. H. Edgeworth, 144, 187.
Gordon, Dr. R. G.—The clinical manifestations and treatment of coliuria in children, 286.
Gordon, Dr. W.—On digital percussion and the cardiac sign in carcinoma, 19.
Grace, Gerald, Obituary notice of, 255.
Gynæcology, The evolution of—Dr. W. H. C. Newnham, 257.

Habershon, Dr. S. H.—The secondary infections of pulmonary tuberculosis, and their treatment by vaccines, 97.
Harty, Dr. J. P. I.—Notes on the electrical reactions in facial paralysis, 55.
Heart, Diet in diseases of the, 217.
Hemorrhages, "Occult," from the stomach and intestines, 220.
Hernia, Case of Richter's strangulated—C. A. Moore, 198.
Hernia, Some unusual conditions found in operating for radical cure of—C. A. Morton, 12.
Hyperpiesis and arterio-sclerosis—Dr. C. W. J. Brasher, 41.

Killian, Professor, visit to Bristol, 177.

Lachrymal obstruction, Operation for, 302.
Laryngeal tuberculosis, Notes on treatment by tuberculin—Dr. P. Watson-Williams, 136.
Leukæmia in a child, Case of lymphatic—Dr. E. C. Williams, 147.
Library of the Bristol Medico-Chirurgical Society, 91, 184, 252, 316; Annual report, 318.
Lighting, Effect of, on the eye, 304.
Liver, Abdominal tumour in the neighbourhood of, 94.
Local medical notes, 96, 192, 256, 320.
Lungs, Diseases of, associated with Friedländer's bacillus—Dr. J. M. Clarke, 1.

Malignant growths of the alimentary canal, "Occult" blood in, 223.
Mastoiditis, Acute, 95.
Medicine, Reports on—Dr. J. M. Clarke, 217; Dr. J. M. Fortescue-Brickdale, 297; Dr. G. Parker, 151; Dr. R. S. Smith, 63.
Mesenteric vessels, Embolism and thrombosis of, 68.
Moore, C. A.—A case of Richter's strangulated hernia, 198.
Morton, C. A.—Some unusual conditions found in operating for the radical cure of hernia, 12.

Nasal pansinusitis, Case of, 319.
Nephritis, Diet in, 219.
Nervous glycosuria, 298.
Newnham, Dr. W. H. C.—Fibroid uterus, 37; The evolution of gynæcology, 257.

Obituary, 189, 253.
"Occult" hemorrhages from the stomach and intestines, 220.
Ogilvy, A., Obituary notice of, 189.
Ophthalmology, Report on—C. H. Walker, 302.

Parker, Dr. G.—Report on medicine, 151.
Percussion, Digital, and the cardiac sign in carcinoma — Dr. W. Gordon, 19.
Pharmacopœia, Some impressions of the 1914 British—Dr. O. C. M. Davis, 292.
Pneumothorax, Artificial, 157.
Poliomyelitis, 155.
Prostatism, 159.
Pulmonary tuberculosis, Drug treatment of, 67.
Pulmonary tuberculosis, Mixed infections in, and some general observations on treatment with tuberculin—Dr. N. Bardswell, 125.
Pulmonary tuberculosis, Secondary infections of, and their treatment by vaccines—Dr. S. H. Habershon, 97.
Pylorus, permeable, Function of gastro-enterostomy opening in, 228.

Radiographic centroscope and the radiographic episcope—Dr. W. Cotton, 202.
Renal glycosuria, 299.
Richter's strangulated hernia, Case of—C. A. Moore, 198.

Salvarsan in ophthalmic practice, 305.
Sanatorium medical staff, Duties of, 64.
Sclerosis, Case of combined, 94.
Smith, Dr. G. M.—Notes on some Bristol medical societies, 268.
Smith, Dr. R. S.—Report on medicine, 63.
Society, Bristol Medico-Chirurgical, 93, 187, 317.
Spiritual healing, 214.
Splenomegaly with anæmia, 151.
Steele, C., Obituary notice of, 253.

Surgery, Reports on—Dr. J. L. Firth, 68, 224; Dr. J. Swain, 159, 300.
Swain, Dr. J.—Reports on surgery, 159, 300.
Syphilis, Cases and specimens of, 93.

Tachycardia, Occurrence of, in association with parenchymatous goitre—Dr. F. H. Edgeworth, 144, 187.
Therapeutic Notes — Dr. J. M. Fortescue-Brickdale, 88, 181, 249.
Tuberculin, Treatment of laryngeal tuberculosis with—Dr. P. Watson-Williams, 136.
Tuberculin, Treatment of mixed infections in pulmonary tuberculosis with—Dr. N. Bardswell, 125.
Tuberculosis officer, Duties of, 64.
Tuberculosis, The campaign against, 63.
Tumour, Abdominal, in the neighbourhood of the liver, 94.

Ulcer, Surgical treatment of gastric, 224.
Uterus, Fibroid—Dr. W. H. C. Newnham, 37.

Vaccines in treatment of secondary infections of pulmonary tuberculosis—Dr. S. H. Habershon, 97.

Walker, C. H.—Report on Ophthalmology, 302.
Walters, C. F.—Autoplastic intramedullary bone pegging as a method of operative treatment for fractures, 139.
War, The, and the Bristol profession, 242, 315.
Watson-Williams, Dr. P.—Notes on the treatment of laryngeal tuberculosis by tuberculin, 136.
Williams, Dr. E. C.—Notes on a case of lymphatic leukæmia in a child, 147.
Winsley sanatorium, Management of, 179, 247.

